# Eye tracking as a tool to quantify the effects of CAD display on radiologists’ interpretation of chest radiographs

**DOI:** 10.1016/j.ejro.2026.100731

**Published:** 2026-01-15

**Authors:** Daisuke Matsumoto, Tomohiro Kikuchi, Yusuke Takagi, Soichiro Kojima, Ryoma Kobayashi, Daiju Ueda, Kohei Yamamoto, Sho Kawabe, Harushi Mori

**Affiliations:** a Department of Radiology, Jichi Medical University, 3311-1 Yakushiji, Shimotsuke-shi, Tochigi 329-0498, Japan; b Medical AI Promotion Institute Co., Ltd., Life Science Building, 12-9 Nihonbashi Odenmacho, Chuo-ku, Tokyo, Japan; c Department of Artificial Intelligence, Graduate School of Medicine, Osaka Metropolitan University, Asahi-machi, Abeno-ku, Osaka 545-8585, Japan; d Center for Health Science Innovation, Osaka Metropolitan University, Asahi-machi, Abeno-ku, Osaka 545-8585, Japan

**Keywords:** Eye-tracking, Concurrent reader, Chest radiography, Bounding-box display, Interpretation time

## Abstract

***Background*:**

Computer-aided detection (CAD) systems for chest radiographs are widely used; however, concurrent reader displays such as bounding-box (BB) highlights may influence interpretation. This pilot study used eye tracking to examine which aspects of visual search were affected by these factors.

***Methods*:**

We sampled 180 chest radiographs from the VinDR-CXR dataset: 120 with solitary pulmonary nodules or masses and 60 without. BBs were configured for 80 % display sensitivity and specificity. Three radiologists (with 11, 5, and 1 years of experience) interpreted each case twice—once with BBs visible and once without—after a ≥ 2-week washout. Eye movements were recorded using an EyeTech VT3 Mini. Metrics included interpretation time, time to first fixation, lesion dwell time, total gaze-path length, and lung-field coverage. Outcomes were modeled using a linear mixed model with the reading condition set as a fixed effect and case and reader as random intercepts. Primary analysis was restricted to true positives (n = 96).

***Results*:**

Concurrent BB display prolonged interpretation time by 4.9 s (p < 0.001) and increased lesion dwell time by 1.3 s (p < 0.001). Total gaze-path length increased by 2076 pixels (p < 0.001), and lung-field coverage increased by 10.5 % (p < 0.001). The time to first fixation was reduced by 1.3 s (p < 0.001).

***Conclusion*:**

Eye tracking revealed measurable changes in search behavior associated with concurrent BB display during chest radiograph interpretation. These findings support this approach and highlight the need for larger studies across modalities and clinical contexts.

## 1 Introduction

Chest radiography (CXR) is one of the most widely performed imaging examinations worldwide and plays a critical role in the early detection of serious diseases, such as lung cancer and pulmonary infections [1]. Small pulmonary nodules are particularly prone to perceptual errors and missed diagnoses, highlighting the need for improved diagnostic accuracy [2].

Computer-aided detection/diagnosis (CAD) for CXR has emerged as a leading application of imaging artificial intelligence (AI), with nodule-detection systems now commercially available [3]. Among these, nodule detection CAD systems are widely available in many countries. Second-reader CAD preserves the opportunity for radiologists to perform habitual systematic searches before exposure to AI findings. In contrast, concurrent reader CAD may alter the search strategy from the beginning of interpretation, potentially changing how abnormalities are located and verified [4].

Previous studies have frequently reported changes in diagnostic accuracy and reading time with CAD/AI assistance [3], [5], [6]. In contrast, few studies have quantitatively assessed how CAD displays affect visual search behavior, including how radiologists initiate their search, verify detected abnormalities, and examine the image [7], [8]. Eye tracking offers objective, fine-grained measurements of visual search processes, including time-to-first fixation, dwell time on the target, total gaze-path length, and coverage of the area of interest [7], [9]. These metrics allow the quantitative assessment of the radiologists’ visual search behavior under different CAD display conditions. Eye tracking research in radiology has characterized search strategies and error types [9]; however, its application in assessing the influence of different CAD display modes, especially concurrent CAD prompts, in CXR interpretation remains limited.

This pilot study aimed to explore whether eye tracking can serve as a useful tool for detecting differences in visual search behavior when radiologists interpret chest radiographs with concurrent-reader–style bounding-box highlighting. Understanding these effects may provide optimal strategies for AI-assisted CXR interpretation in clinical practice.

## 2 Materials and methods

### 2.1 Case distribution

**Fig. 1:**
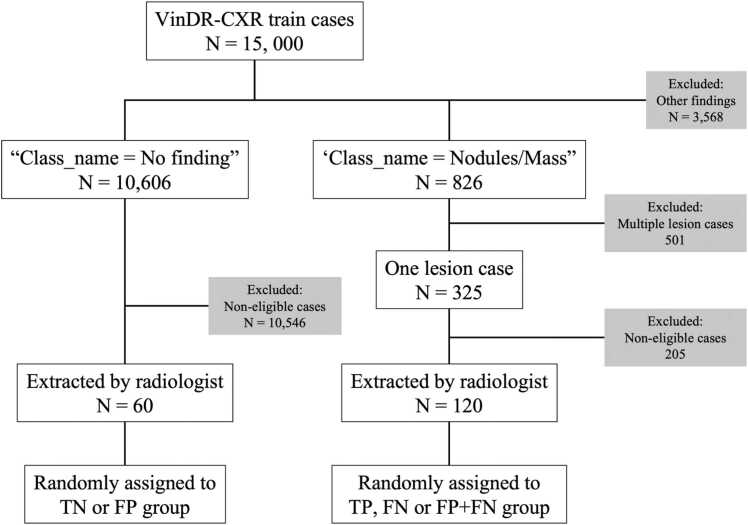
Case selection flows from the VinDR-CXR dataset.

Images used in this study were drawn from the VinDR-CXR dataset [10]. Fig. 1 illustrates the case selection flow. A total of 180 chest radiographs were included. Among the selected images, 120 contained a solitary pulmonary nodule or mass and were randomly allocated into the following groups: (i) true-positive (TP), lesion-positive with a bounding box (BB) correctly marking the lesion location (n = 96); (ii) false-negative (FN), lesion-positive with no BB on the lesion and no incorrect BB elsewhere (n = 12); and (iii) FN plus false-positive (FP), lesion-positive with no BB on the lesion but with an incorrect BB elsewhere (n = 12). The remaining 60 lesion-negative images were randomly divided into (iv) true-negative (TN) with no BB (n = 48) and (v) FP images with a manually placed incorrect BB (n = 12). This allocation yielded an overall BB-display sensitivity of 96/120 (80 %) and specificity of 48/60 (80 %). For the TP cases, BBs were adopted from the VinDR-CXR ground-truth annotations, whereas for the FP cases, a radiologist manually placed BBs on structures commonly misinterpreted as pulmonary lesions, such as rib overlaps and nipple shadows.

### 2.2 Reader study

Two experimental conditions were tested: with and without CAD assistance. A crossover design was employed with each reader interpreting the same cases under both conditions. In session 1, the readers interpreted all 180 cases under a simulated concurrent reader CAD setting; the BBs were displayed according to the predefined groups and placements described above. In session 2, after a washout period of at least 2 weeks to minimize recall bias [11], the same 180 cases were re-read without CAD assistance, with the case order randomized for each session. Before the experiment, a separate set of 10 cases was used as a practice session to familiarize the readers with the graphical user interface. These practice cases were excluded from the subsequent analyses. No feedback regarding the correctness of the interpretations was provided in any of the sessions.

Three radiologists (with 11, 5, and 1 year of experience) participated in the study. The readers were instructed to assess only lung fields. They were informed that the BBs represented AI outputs with nominal specifications of 80 % sensitivity and 80 % specificity; however, as described above, these BBs were manually created to achieve these figures. Readers were notified that eye-tracking would be performed, but the specific purpose was not disclosed.

### 2.3 Eye-tracking data acquisition

**Fig. 2:**
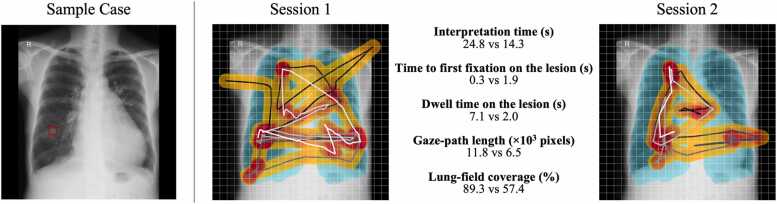
Example of eye-tracking visualization. The cyan overlay masks the lung fields; the gaze path starts in black and ends in white, and a 50-pixel buffer around the path is shown as an orange band, whose overlap density is visualized as a heat map graduating from orange to red.

The eye movements were recorded at a sampling rate of 60 Hz using a VT3 Mini device (EyeTech Digital Systems; Mesa, AZ, USA). Images were displayed on a 27-inch FlexScan EV2781 monitor (EIZO Corporation; Ishikawa, Japan) with a resolution of 2560 × 1440 pixels, a pixel pitch of 0.233 mm, and a maximum brightness of 350 cd/m². The distance between the participant’s eyes and the screen was approximately 60 cm. To ensure high spatial accuracy, a 16-point calibration procedure was performed for each participant immediately before every data collection session. The following metrics were extracted: (i) Total interpretation time; (ii) Dwell time on lesion: the cumulative duration during which the gaze remained within the lesion area, defined as the lesion BB plus a 50-pixel margin. Under these experimental conditions, this 50-pixel margin corresponds to a visual angle of approximately 1.1 °. This threshold was chosen to encompass the foveal vision span (typically 1–2 °) and is consistent with thresholds used in recent eye-tracking studies in radiology [8]; (iii) Time to first fixation on lesion: the elapsed time from the start of case interpretation until the gaze first entered the defined lesion area; (iv) Total gaze-path length: the sum of gaze trajectory lengths measured in pixels; and (v) Lung-field coverage ratio—calculated as the proportion of 50-pixel lung-field grid cells crossed by the gaze path (incorporating the 50-pixel margin to reflect foveal vision). Lung fields were generated using a deep-learning-based model (ChestXRayAnatomySegmentation; https://github.com/ConstantinSeibold/ChestXRayAnatomySegmentation). A board-certified radiologist (with 10 years of experience), who did not participate in the reader study, visually inspected the segmentation results for all cases to confirm that no major corrections were necessary, and the output was used without modification. Fig. 2 shows an example of the gaze visualization.

### 2.4 Statistical analysis

Accuracy was calculated for each reader and each group, and the sensitivity and specificity were computed for each reader across all cases. The primary analysis was limited to the TP group to isolate the effect of concurrent bounding-box display on visual behavior during successful detection. The other case groups (FN, FN+FP, TN, and FP) were included to simulate a realistic reading environment—preventing reader anticipation of lesion presence or AI correctness—rather than for the direct comparison of gaze metrics. Eye-tracking data were considered valid if more than 50 % of gaze samples were successfully captured during the interpretation period of each case. This threshold was chosen to accommodate natural gaze behaviors inherent to the task—such as looking at hands or peripheral UI elements—which may cause temporary tracking interruptions, comparable to exclusion criteria in other eye-tracking studies [12]. Only cases with valid eye-tracking data in both sessions were included in the subsequent linear mixed model (LMM) analyses of eye-tracking metrics, whereas all interpretations were retained for the assessment of diagnostic performance. For each outcome metric, an LMM was fitted with the reading condition (session 1 vs. session 2) as a fixed effect, and the case and reader as random intercepts. Model diagnostics were performed to verify the assumptions of homoscedasticity and normality (see Appendix 1 for diagnostic plots and detailed assessment). Statistical significance was set at p < 0.05. The computational environment was Python 3.11 with the following key packages: Statsmodels 0.14.5, SciPy 1.16.1, NumPy 2.3.2, and Pandas 2.3.1.

### 2.5 Ethics

The use of the open-access VinDR-CXR dataset did not require Institutional Review Board (IRB) approval for patient data. Regarding the reader study, formal IRB approval was not required given the non-invasive nature of the experiment involving volunteer medical professionals. However, the study was conducted in accordance with the ethical standards of the Declaration of Helsinki. Informed consent regarding the recording of eye movements and the use of data was obtained from all participating radiologists.

## 3 Results

Table 1 summarizes the interpretation accuracy and BB adoption rates stratified by the reader and case groups. In the TP group, the accuracy ranged from 63.5 % to 82.3 % in session 2 and from 83.3 % to 99.0 % in session 1, with all readers demonstrating improved accuracy when the BBs were displayed. Across all readers, sensitivity ranged from 60.0 % to 79.2 % and specificity from 63.3 % to 96.7 % in session 2, compared with a sensitivity of 79.2 %–80.8 % and specificity of 76.7 %–86.7 % in session 1.

**Table 1:** Diagnostic performance and bounding-box adoption rates by reader and case group.

**Metric**	**Group**	**Radiologist 1**		**Radiologist 2**		**Radiologist 3**	
		**Session 1**	**Session 2**	**Session 1**	**Session 2**	**Session 1**	**Session 2**
Accuracy (%)	TP	83.3	69.8	99.0	82.3	85.4	63.5
Improved_count*	TP	18		17		25	
Worsened_count**	TP	5		1		4	
BB adoption rate (%)	TP	83.3		99.0		85.4	
Accuracy (%)	FN	66.7	58.3	16.7	66.7	58.3	41.7
Improved_count	FN	1		0		3	
Worsened_count	FN	0		6		1	
BB adoption rate (%)	FN	33.3		75.0		41.7	
Accuracy (%)	FP+FN	79.2	79.2	16.7	62.5	70.8	70.8
Improved_count	FP+FN	1		0		3	
Worsened_count	FP+FN	1		11		3	
BB adoption rate (%)	FP+FN	4.2		33.3		4.2	
Accuracy (%)	FP	91.7	100.0	16.7	58.3	83.3	100.0
Improved_count	FP	0		0		0	
Worsened_count	FP	1		5		2	
BB adoption rate (%)	FP	8.3		66.7		8.3	
Accuracy (%)	TN	72.9	93.8	97.9	64.6	87.5	95.8
Improved_count	TN	1		16		1	
Worsened_count	TN	11		0		5	
BB adoption rate (%)	TN	72.9		97.9		87.5	
BB adoption rate (%)	Total	71.4		94.6		77.4	
Sensitivity (%)	Total	80.8	67.5	80.8	79.2	79.2	60.0
Specificity (%)	Total	76.7	95.0	81.7	63.3	86.7	96.7

BB: Bounding-box

* Improved_count: Number of cases where the diagnostic result changed from incorrect in Session 2 (without BB display) to correct in Session 1 (with BB display).

** Worsened_count: Number of cases where the diagnostic result changed from correct in Session 2 to incorrect in Session 1.

Table 2 presents eye-tracking metrics by reader, along with overall comparisons derived from the LMM. For Radiologist 1, valid eye-tracking data were successfully captured in 96/96 cases (100 %) in Session 1 and 74/96 cases (77.1 %) in Session 2. For Radiologist 2, data were captured in 95/96 cases (99.0 %) in Session 1 and 80/96 cases (83.3 %) in Session 2. For Radiologist 3, data were captured in 94/96 cases (97.9 %) in Session 1 and 96/96 cases (100 %) in Session 2. Based on our listwise deletion criteria (requiring valid data in both sessions), the final number of paired cases included in the LMM analysis was 74 (77.1 %) for Radiologist 1, 79 (82.3 %) for Radiologist 2, and 94 (97.9 %) for Radiologist 3. Under the session 1 condition, interpretation time increased by a mean of 4.9 s (95 % CI [3.3, 6.5], p < 0.001). Dwell time on the lesion increased by 1.3 s (95 % CI [0.6, 1.9], p < 0.001). Time to first fixation on the lesion decreased by 1.3 s (95 % CI [–2.1, –0.5], p < 0.001). Gaze-path length increased by 2076 px (95 % CI [1337, 2816], p < 0.001). Lung-field coverage ratio increased by 10.5 % (95 % CI [8.6, 12.4], p < 0.001).

**Table 2:** Eye-tracking metrics by reader and overall comparisons based on linear mixed model analysis.

Metric	Radiologist 1		Radiologist 2		Radiologist 3		Diff (95 % CI)	p
	Session 1* (96/96)	Session 2** (74/96)	**Session 1** **(95/96)**	**Session 2** **(80/96)**	**Session 1** **(94/96)**	**Session 2** **(96/96)**		
**Valid pairs (N)**	74		79		94			
**Interpretation time** **(sec)**	14.6 [11.7, 22.1]	10.2 [7.7, 13.2]	5.2 [3.5, 7.9]	4.4 [2.8, 7.6]	13.2 [7.9, 25.6]	8.6 [4.1, 15.2]	4.9 (3.3 – 6.5)	< 0.001
**Dwell time on lesion** **(sec)**	2.2 [1.0, 3.7]	1.2 [0.5, 2.1]	1.1 [0.5, 2.1]	0.4 [0.0, 0.9]	3.3 [1.6, 6.7]	1.3 [0.5, 3.7]	1.3 (0.6 – 1.9)	< 0.001
**Time to first fixation** **(sec)**	1.0 [0.7, 2.4]	1.9 [1.0, 7.0]	1.8 [0.9, 3.5]	2.7 [1.2, 5.0]	0.7 [0.6, 1.4]	1.4 [0.9, 3.8]	-1.3 (-2.1 – -0.5)	< 0.001
**Gaze-path length** **(pixels)**	8459 [6334, 12272]	5706 [4447, 9242]	2764 [2028, 3547]	2308 [1514, 3851]	8383 [5692, 12180]	4782 [3034, 7655]	2076 (1337 – 2816)	< 0.001
**Lung-field coverage** **(%)**	85.5 [76.9, 88.5]	68.0 [61.3, 77.7]	52.9 [42.5, 62.6]	46.8 [37.2, 56.7]	73.9 [63.3, 81.2]	62.4 [52.7, 72.7]	10.5 (8.6 – 12.4)	< 0.001

Values are presented as medians [interquartile range, 25th–75th percentile].

* Session 1: Interpretation with bounding-box (BB) display (simulating a concurrent reader CAD setting). Numbers in parentheses for each session indicate the count of cases with valid eye-tracking data out of the 96 true-positive (TP) cases

** Session 2: Interpretation without BB display, conducted after a ≥ 2-week washout period.

## 4 Discussion

In this pilot reader study, eye tracking revealed measurable differences in interpretation time, lesion dwell time, time to first fixation, gaze-path length, and lung-field coverage under a concurrent BB display. These metrics provide a useful means for characterizing how CAD presentation modes influence radiologists’ visual search behaviors during chest radiographic interpretation.

Previous studies have investigated the effects of second-reader CAD on diagnostic performance and reading time. However, the reported impact varies across modalities, diseases, and imaging settings, and a consistent picture has not emerged [13], [14]. Because eye tracking offers the possibility of quantitatively assessing such CAD-related effects, recent studies have begun to apply it in this context. In a multicenter mammography study, Gommers et al. explicitly monitored readers’ eye movements to compare performance and search patterns with and without AI decision support and reported significant differences in gaze behavior: fixation time within lesion regions increased, while overall coverage of the breast area decreased, alongside an improvement in diagnostic accuracy. In the present study, using chest radiographs, interpretation time was prolonged under concurrent BB display, and gaze metrics shifted in a different manner from those reported by Gommers et al. [8]. Time to lesion localization was shortened, dwell time on the lesion increased, and overall lung-field coverage expanded. Taken together, the results indicate a shift toward a “find fast, verify thoroughly” reading pattern under concurrent prompting in the context of chest radiograph interpretation. These contrasts indicate that the influence of AI prompts on visual search behavior may differ according to modality and task, highlighting the importance of context-specific evaluation.

Attention to inter-reader variability is also important, as recent studies have shown heterogeneous impacts of AI-assistance across radiologists and tasks, and even experienced readers may remain susceptible to AI-driven influences such as automation bias [15], [16]. In this study, the BB adoption rate (Table 1) and gaze metrics (Table 2) indicated inter-reader differences, suggesting differing degrees of reliance on BB prompts. Thus, eye-tracking may provide a means to characterize reader-specific patterns of AI utilization. Although detailed inter-reader comparisons or typological classifications are beyond the scope of this pilot study, such analyses represent an important direction for future research and underscore the need for users to understand the specifications and performance characteristics of the products they deploy and to adapt their workflows accordingly.

As demonstrated in previous studies and this pilot investigation, the chosen presentation mode (e.g., second reader vs. concurrent reader) may influence radiologists’ reading time and behavior. Therefore, it is desirable for product information to indicate how such modes could affect the reading process and how these effects are intended to be handled in practice. For products already in clinical use or those to be introduced in the near future, radiologists should be made aware of these potential effects, and CAD providers should bear the responsibility of communicating such information transparently. Recent societal statements also emphasize the importance of transparent information transfer from the provider to the deployer and clear user guidance regarding AI tools in radiology [17].

### 4.1 Limitations and future directions

This study had certain limitations. First, as this was a pilot study primarily aimed at demonstrating the feasibility of eye-tracking metrics in this context, a formal a priori power analysis for the linear mixed model was not performed, and the sample size was determined based on feasibility. Consequently, the statistical power to detect small effects may be limited. Second, only three readers participated; thus, conclusions regarding the direction of inter-reader variability remain limited, and statistical estimates of this variability should be interpreted with caution. Third, this study employed a simulated CAD scenario. Although the BBs for the TP group were adopted from the ground-truth annotations of the original dataset, the BBs for the FP group were manually configured and may not reflect the full error distribution or confidence characteristics of real-world production AI systems. Fourth, the primary analysis was restricted to TP cases, and behavior under FP prompting was not evaluated (descriptive summaries of other case groups are provided in Appendix 2). Fifth, the application of a strict validity threshold (requiring >50 % gaze capture in both sessions) resulted in the exclusion of a subset of eye-tracking data. Notably, the exclusion rates were higher for Radiologists 1 and 2, particularly during Session 2. Although we employed listwise deletion to ensure that the LMM analysis was conducted on an identical set of cases across both conditions for each reader, this reduction in the number of valid paired cases may have influenced the statistical power for detecting subtle differences in gaze behavior and could potentially introduce a bias in the comparison.

To address these limitations, we plan to expand the scale of the experiment and conduct it in the context of real-world AI use. We will also increase the number of readers to enable a more systematic evaluation of inter-reader variability and investigate the relationship between accuracy and eye-tracking metrics to gain further insight into how AI-assistance influences both performance and visual search behavior.

### 4.2 Conclusion

This pilot study demonstrated that eye tracking can capture how the presence or absence of CAD influences radiologists’ interpretation of chest radiographs, identifying measurable changes across interpretation time, lesion dwell time, time to first fixation, gaze-path length, and lung-field coverage. These findings further suggest that reader responses to AI prompts vary across modalities, underscoring the need for broader context-specific investigations.

## Funding statement

This research did not receive any specific grant from funding agencies in the public, commercial, or not-for-profit sectors.

## Ethical statement

As only publicly available data were used, Institutional Review Board (IRB) approval was not required for the imaging data. Regarding the reader study, formal IRB approval was not required given the non-invasive nature of the experiment involving medical professionals; however, the study was conducted in accordance with the Declaration of Helsinki. Informed consent regarding the recording of eye movements and the use of data was obtained from all participating radiologists.

## CRediT authorship contribution statement

**Sho Kawabe:** Writing – review & editing. **Kohei Yamamoto:** Writing – review & editing. **Harushi Mori:** Writing – review & editing. **Tomohiro Kikuchi:** Writing – original draft, Visualization, Supervision, Software, Project administration, Methodology, Investigation, Formal analysis, Data curation, Conceptualization. **Daisuke Matsumoto:** Writing – original draft. **Soichiro Kojima:** Writing – review & editing. **Yusuke Takagi:** Writing – review & editing, Visualization. **Daiju Ueda:** Writing – review & editing. **Ryoma Kobayashi:** Writing – review & editing.

## Declaration of Competing Interest

The authors declare the following financial interests/personal relationships which may be considered as potential competing interests: Tomohiro Kikuchi, Yusuke Takagi, Daiju Ueda, Sho Kawabe report a relationship with Medical AI Promotion Institute that includes: board membership and employment. If there are other authors, they declare that they have no known competing financial interests or personal relationships that could have appeared to influence the work reported in this paper.
