# Supplementary material for: Eye tracking as a tool to quantify the effects of CAD display on radiologists’ interpretation of chest radiographs
Source: Eur J Radiol Open. 2026 Jan 15;16:100731. doi: 10.1016/j.ejro.2026.100731 (PMC12834923; doi:10.1016/j.ejro.2026.100731)
Supplement: Supplementary file 1 — Supplementary material [file mmc1.pdf]

Appendix 1. Diagnostic plots for linear mixed model (LMM) residuals.

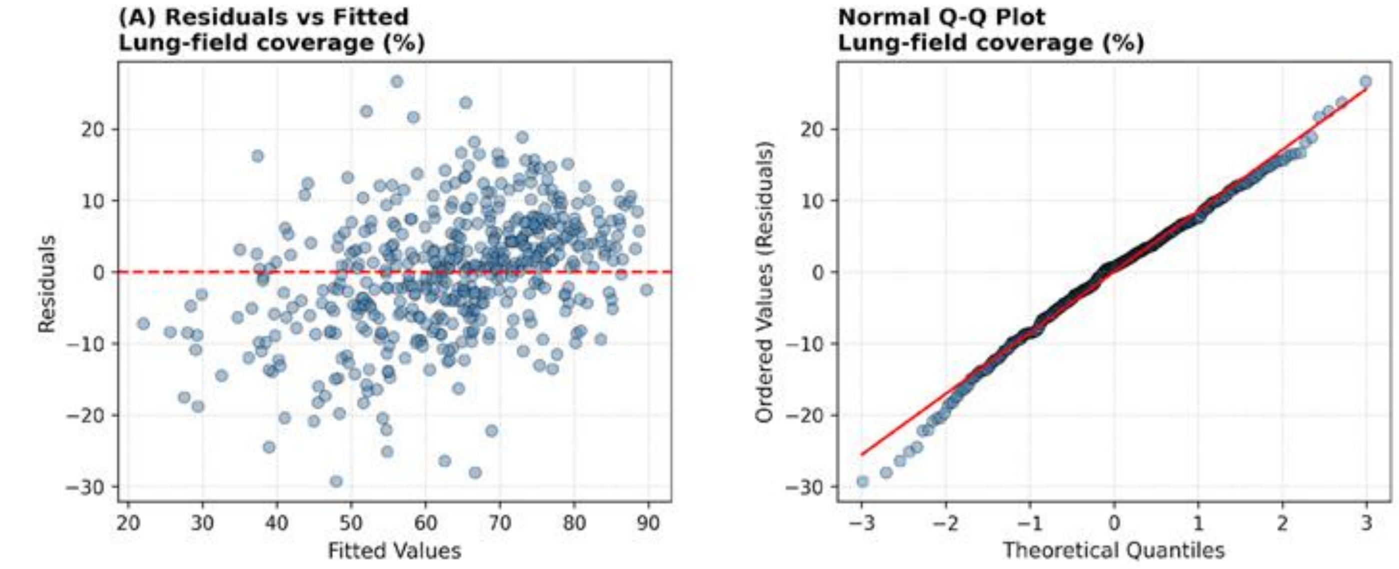

(A) Residuals vs. Fitted plot (left) and Normal Q-Q plot (right) for Lung-field coverage ratio (%). The plots demonstrate good adherence to the assumptions of homoscedasticity and normality, with data points closely following the theoretical reference line in the Q-Q plot.

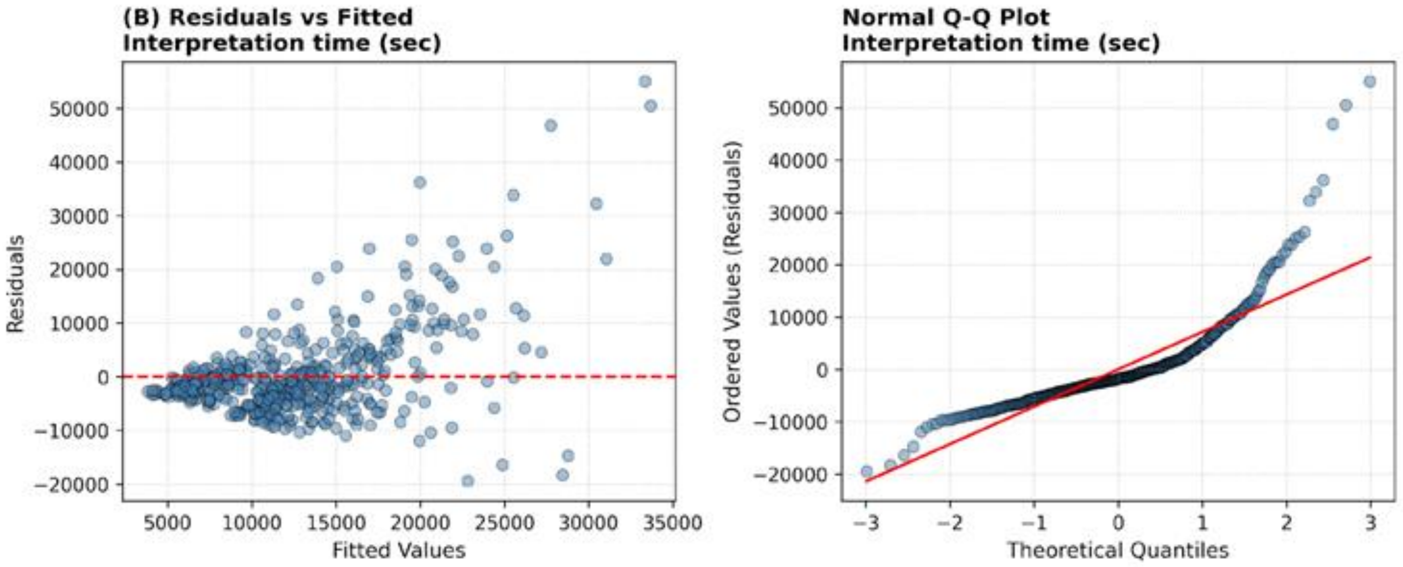

(B) Residuals vs. Fitted plot (left) and Normal Q-Q plot (right) for Interpretation time (sec). The Q-Q plot exhibits a characteristic deviation in the upper tail, reflecting the right-skewed nature of time-based data. However, the raw data were retained for analysis to preserve clinical interpretability (i.e., seconds), relying on the relative robustness of LMMs to moderate deviations from normality.

Appendix 2. Descriptive summary of eye-tracking metrics for all case categories and individual readers.

Metric	Group	Radiologist 1			Radiologist 2			Radiologist 3		
		Session1	Session2	Valid cases	Session1	Session2	Valid cases	Session1	Session2	Valid cases
Interpretation time (sec)	TP	10.2	14.6	74	4.4	5.2	79	8.6	13.2	94
	FN	13.4	21.4	10	5	11.7	8	8.5	22.7	12
	FP+FN	13	21.6	21	9.8	10.7	18	9.3	20.9	23
	FP	12.2	21.6	11	11.7	9.3	10	10.1	20.9	11
	TN	10.4	17.6	35	16.8	4.1	40	9.9	18.5	48
Gaze path length (pixels)	TP	5706	8459	74	2308	2764	79	4782	8383	94
	FN	8012	8608	10	2155	4026	8	4395	10129	12
	FP+FN	7438	9399	21	3079	4128	18	5504	8575	23
	FP	7438	10097	11	4566	4409	10	6201	8575	11
	TN	7319	10160	35	4759	2230	40	5757	10511	48
Lung-field coverage (%)	TP	68	85.5	74	46.8	52.9	79	62.4	73.9	94
	FN	77.9	86	10	41.4	61.6	8	67.3	68.9	12
	FP+FN	72.4	87.1	21	51.9	59.3	18	70.7	75.2	23
	FP	65.1	88.2	11	56.8	56.9	10	75.1	75.7	11
	TN	74.8	84.1	35	58.3	50.5	40	72.8	84.2	48
